# Epigenetic biomarkers of borderline personality disorder with severe suicidal behaviors

**DOI:** 10.1192/j.eurpsy.2024.86

**Published:** 2024-08-27

**Authors:** J. Jokinen

**Affiliations:** ^1^Clinical sciences, Umeå University, Umeå; ^2^Clinical neuroscience, Karolinska Institutet, Stockholm, Sweden

## Abstract

**Abstract:**

Borderline personality disorder (BPD) is associated with excess suicide risk, natural-cause mortality, comorbid medical conditions, poor health habits and stress related epigenomic alterations. This presentation will report findings of *BDNF* and stress system associated epigenetic alterations in a group of severely impaired BPD and suicidal patients. Further, findings of GrimAge – a state-of-the-art epigenetic age (EA) estimator- in patients with BPD and attempted suicide patients will be presented. Genome-wide methylation patterns were measured using the Illumina Infinum Methylation Epic BeadChip in whole blood from well characterized 97 BPD patients, 88 suicide attempters and 32 healthy controls.

**Disclosure of Interest:**

None Declared

